# A comparison of ARMS-Plus and droplet digital PCR for detecting EGFR activating mutations in plasma

**DOI:** 10.18632/oncotarget.22997

**Published:** 2017-12-06

**Authors:** Xinxin Zhang, Ning Chang, Guohua Yang, Yong Zhang, Mingxiang Ye, Jing Cao, Jie Xiong, Zhiping Han, Shuo Wu, Lei Shang, Jian Zhang

**Affiliations:** ^1^ Department of Pulmonary Medicine, Xijing Hospital, Fourth Military Medical University, Xi’an, China; ^2^ Department of Health Statistics, School of Preventive Medicine, Fourth Military Medical University, Xi'an, China; ^3^ Research and Development Department, GenoSaber Biotech Co. Ltd., Shanghai, China

**Keywords:** NSCLC, EGFR, liquid biopsy

## Abstract

In this study, we introduce a novel amplification refractory mutation system (ARMS)-based assay, namely ARMS-Plus, for the detection of epidermal growth factor receptor (EGFR) mutations in plasma samples. We evaluated the performance of ARMS-Plus in comparison with droplet digital PCR (ddPCR) and assessed the significance of plasma EGFR mutations in predicting efficacy of EGFR-tyrosine kinase inhibitor (TKI) regimen. A total of 122 advanced non-small cell lung cancer (NSCLC) patients were enrolled in this study. The tumor tissue samples from these patients were evaluated by conventional ARMS PCR method to confirm their EGFR mutation status. For the 116 plasma samples analyzed by ARMS-Plus, the sensitivity, specificity, and concordance rate were 77.27% (34/44), 97.22% (70/72), and 89.66% (104/116; κ=0.77, *P*<0.0001), respectively. Among the 71 plasma samples analyzed by both ARMS-Plus and ddPCR, ARMS-Plus showed a higher sensitivity than ddPCR (83.33% versus 70.83%). The presence of EGFR activating mutations in plasma was not associated with the response to EGFR-TKI, although further validation with a larger cohort is required to confirm the correlation. Collectively, the performance of ARMS-Plus and ddPCR are comparable. ARMS-Plus could be a potential alternative to tissue genotyping for the detection of plasma EGFR mutations in NSCLC patients.

## INTRODUCTION

Targeted therapy has opened a new era for cancer treatment. Indeed, epidermal growth factor receptor tyrosine kinase inhibitors (EGFR-TKIs), have been emerged as the first-line therapy for advanced non-small cell lung cancer (NSCLC). EGFR-TKIs significantly prolong the survival and improve the quality of life of patients with advanced NSCLC. However, they are only effective to a specific subtype of patients harboring EGFR activating mutations such as in-frame deletions of exon 19 (19del) and L858R substitution in exon 21 (L858R) [1–5]. Therefore, a reliable genotyping assay is required to screen for patients who would benefit from the EGFR-TKI regimens.

Currently, tissue biopsy remains to be the standard diagnostic procedure for EGFR genotyping. Despite its reliability, it is difficult to collect sufficient tissue samples for molecular analysis. A previous study reported that only 48% of all the biopsy fragments derived from bronchial biopsy contain tumor [6]. The low quantity of tumor severely limited the application of tissue specimens in clinical practice. In addition, a biopsy section obtained from a single part of a solitary tumor cannot reflect the full genomic landscape of tumor heterogeneity [7]. This would lead to false-negative results and affect clinical decision making. Recently, circulating tumor DNA (ctDNA), as a potential alternative to tissue biopsy, has become a hot topic in cancer research. ctDNA are DNA fragments shed from the primary or metastatic tumors and entered the blood circulation. Liquid biopsies based on ctDNA are non-invasive and thus allow repetitive and longitudinal monitoring of the tumor evolution. More importantly, it overcomes the challenge of tumor heterogeneity [8, 9].

Nevertheless, the majority of currently available ctDNA-based genotyping assays possess a low sensitivity and are unable to quantify mutations. These greatly hampered their use for molecular analysis [10]. For example, the amplification refractory mutation system (ARMS)-based techniques showed a relatively poor sensitivity of 45.4–65.7% in a real-world setting [5, 11]. To improve the detection sensitivity, quantitative digital platforms have been developed [12]. These include beads, emulsions, amplification and magnetics (BEAMing) and droplet digital polymerase chain reaction (ddPCR), which have been demonstrated to be highly sensitive in the detection of plasma EGFR mutations [13, 14]. The mechanism of ddPCR is to compartmentalize DNA samples into water-oil emulsion droplets and amplify each DNA fragment as a single molecule. Hence, it enables an absolute count of the target mutations present in the samples. According to the most updated prospective study, the sensitivity of plasma ddPCR for EGFR 19del and L858R were 82% and 74% respectively, and the specificity were 100% for both mutations [8].

Despite the improvement in diagnostic performance, ddPCR requires unique equipment for sample processing and analysis. This complicates the analytical process and increases the cost of the assay. Herein, we introduce a novel method based on ARMS PCR, namely ARMS-Plus, for the detection of EGFR mutations. ARMS-Plus can be performed simply with a real-time PCR device. Conventional ARMS PCR technology suffers from a high rate of false-positive due to the non-specific binding of primers to the wild-type DNA. To solve this problem, we employed a “wild-type blocker” to the PCR reaction pool, which is complementary to the wild-type DNA at the mutation sites (Supplementary Figure 1). The annealing of the “Wild-type blocker” prohibits the non-specific amplification and thus increases the detection specificity. Moreover, the amplicon of each ARMS-Plus reaction was shortened to 50-80 bp. This feature enables ARMS-Plus to adapt to the highly fragmented DNA extracted from the plasma and thus enhances its detection sensitivity. According to our study, ARMS-Plus can reliably detect EGFR mutations with a detection limit of at least 0.015%. Additionally, ARMS-Plus is capable of quantifying EGFR mutations. This provides additional information for guiding the treatment decision in NSCLC.

The aim of this study was to evaluate the performance of ARMS-Plus in detecting plasma EGFR mutations. In addition, we conducted a head-to-head comparison between ARMS-Plus and ddPCR in detecting EGFR activating mutations. We also investigated the association of plasma EGFR mutation status with clinical responses to EGFR-TKIs in advanced NSCLC patients.

## RESULTS

### Characterization of ARMS-Plus

Mutant-specific primers were meticulously designed to detect EGFR L858R and two types of 19del, E746_A750del (1) (19del 1) and E746_A750del (2) (19del 2), which account for nearly 80% of total 19del cases [15]. Detection of either deletion will be regarded as EGFR 19del positive.

**Figure 1:**
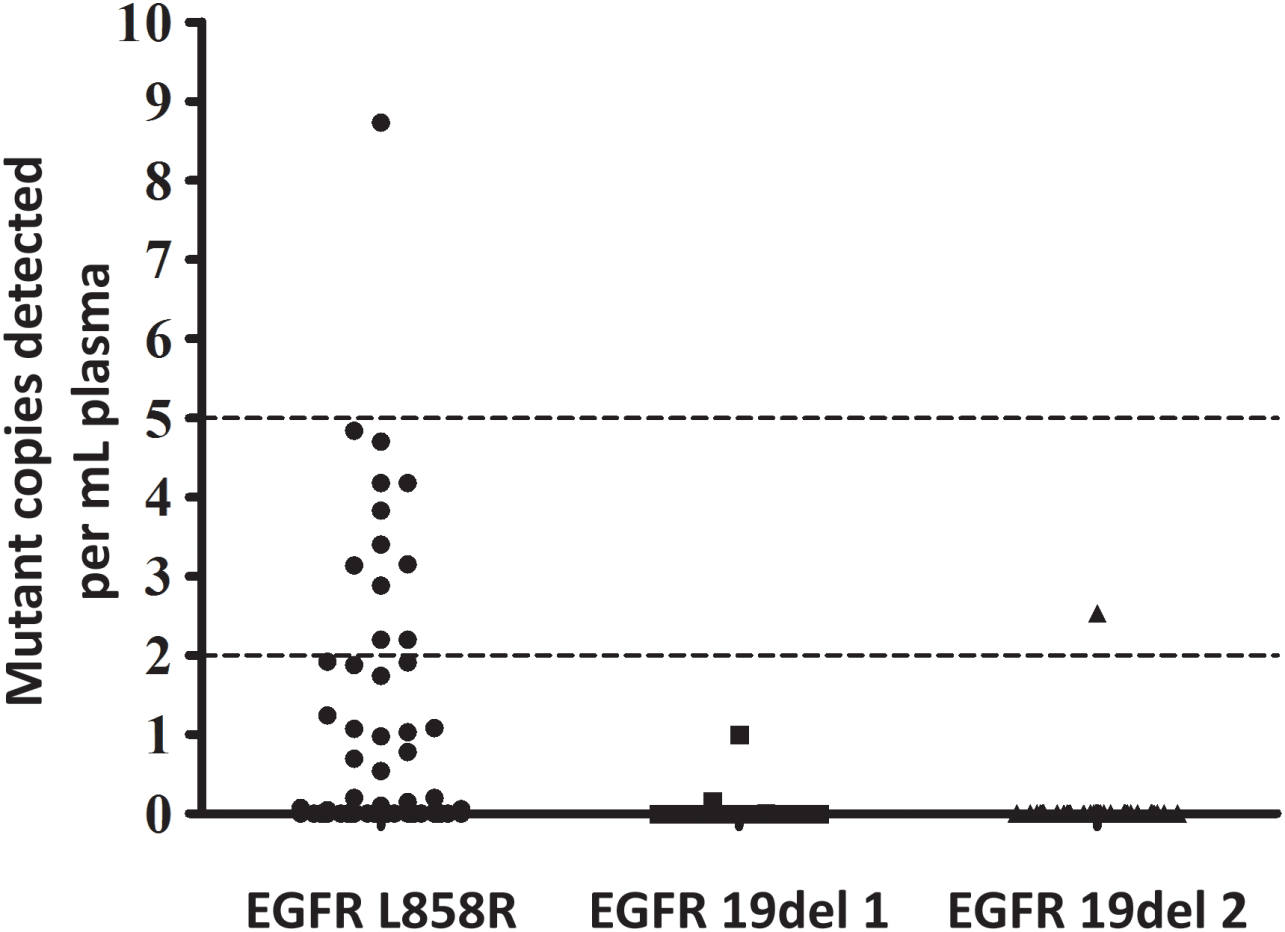
Determination of detection limit of ARMS-Plus 3 or 10 corresponding EGFR mutant copies in a background of 20,000 copies wt gDNA were tested by ARMS-Plus, with pure wt gDNA as a negative control. The mutations detected per reaction were plotted with the box chart. Both EGFR 19del 1, 19del 2, and L858R mutations were stably detected by ARMS-Plus. The detection limit of ARMS-Plus is at least 0.015%.

To establish a legitimate system for the evaluation of the analytical sensitivity of ARMS-Plus, EGFR mutant plasmids were diluted with wild-type genomic DNA (wt gDNA) extracted from leukocytes of healthy individuals. For each type of mutation, 3 or 10 copies of EGFR mutant plasmids were spiked into 20,000 copies of wt gDNA, with pure wt gDNA as a negative control. Each reaction was performed in quadruplicate. Results showed that ARMS-Plus can stably detect all mutations in the spiked samples with 0.015% (3/20,000) and 0.05% (10/20,000) mutation rates (Figure 1). Thus the detection limit of ARMS-Plus is determined to be at least 0.015%.

**Figure 2:**
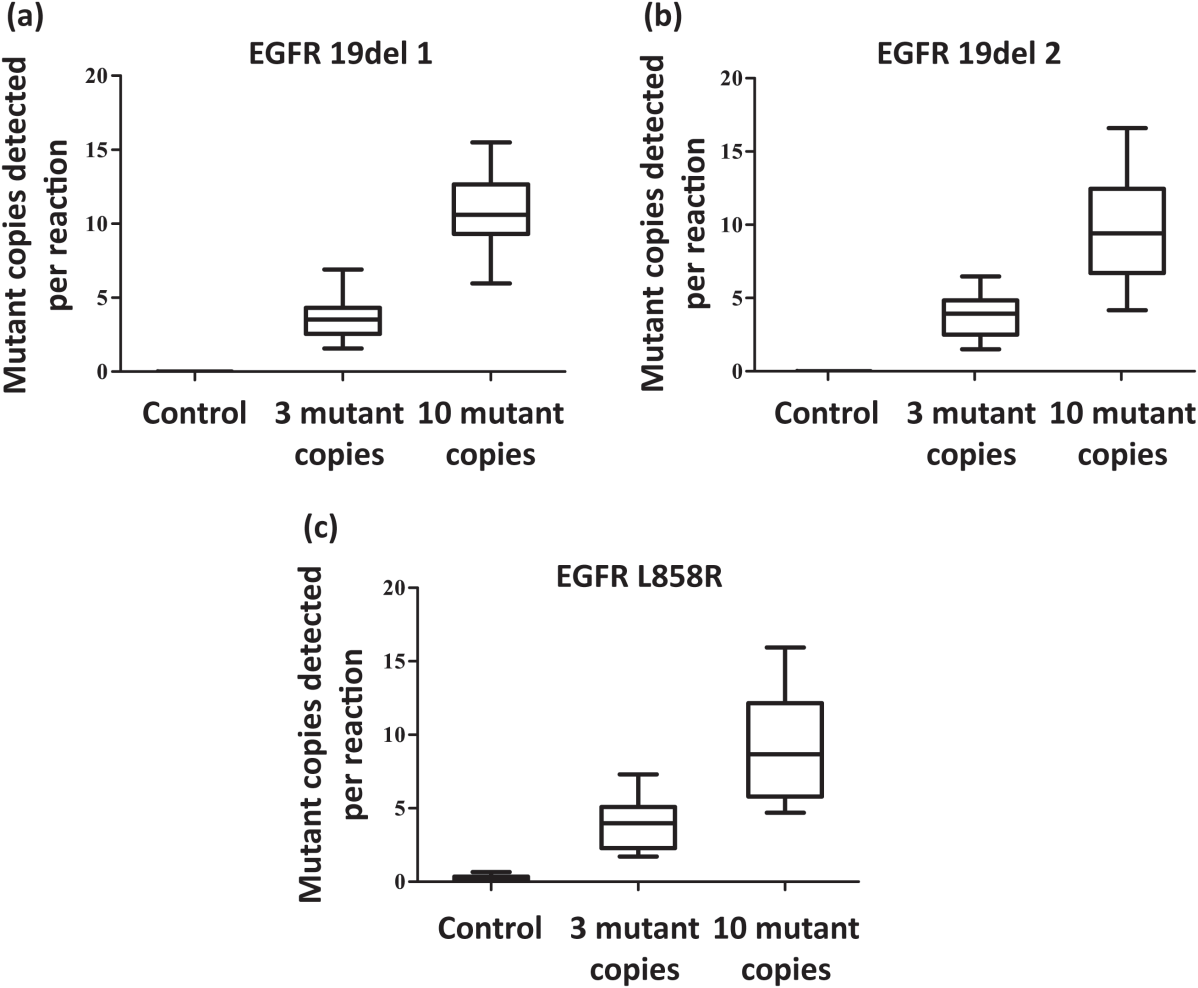
Determination of cut-off values for EGFR mutations Background EGFR mutations presented in the plasma cfDNA of 112 healthy individuals were evaluated by ARMS-Plus. The cut-off values for L858R and 19del were 5 copies/mL and 2 copies/mL, respectively.

Moreover, background EGFR mutations have been reported to present in the circulating cell-free DNA (cfDNA) of healthy individuals [16]. To standardize the definition of mutations detected by ARMS-Plus in routine clinical practice, we obtained plasma samples from 112 healthy individuals and evaluated the abundance of EGFR mutation in cfDNA. Results demonstrated that L858R mutant abundance was less than 5 copies/mL in almost all of the plasma samples, except for only one case of 8.7 copies/mL. For the two 19del mutations, the highest concentration detected was 1.1 and 2.8 copies/mL, respectively. As a result, we define the cut-off values of EGFR L858R and 19del as 5 copies/mL (Figure 2a, 2b) and 2 copies/mL (Figure 2c), respectively.

### Patient demographics

A total of 122 newly-diagnosed, treatment-naïve advanced NSCLC patients were enrolled in this study (Table 1). All patients underwent biopsy for tissue genotyping with conventional ARMS PCR method. Of these patients, 46.7% (57/122) were female, median age was 59 years (range from 37-91 years), and 58.2% (71/122) were never-smokers. The majority of these patients (81.1%, 99/122) were diagnosed with lung adenocarcinoma. All of the patients were diagnosed with advanced stage NSCLC (IIIa: 5.7%, IIIb: 29.5%, IV: 64.8%).

**Table 1:** Patient demographics

	n (%)
Patients no.	122
Age	
Median (Range)	59 (30-85)
Gender	
Male	65 (53.3)
Female	57 (46.7)
Smoking status	
Never smoker	71 (58.2)
Smoker	51 (41.8)
Histologic type	
Adenocarcinoma	99 (81.1)
Squamous cell carcinoma	23 (18.9)
Stage	
IIIa	7 (5.7)
IIIb	36 (29.5)
IV	79 (64.8)
Performance status	
0-2	115 (94.3)
3-4	7 (5.7)
EGFR mutation status (by tissue genotyping)	
EGFR activating mutation positive	45 (36.9)
EGFR activating mutation negative	77 (63.1)
Received EGFR-TKIs treatment	44 (36.1)
Objective response rate	18/44 (40.9)
Disease control rate	41/44 (93.2)

**Figure 3:**
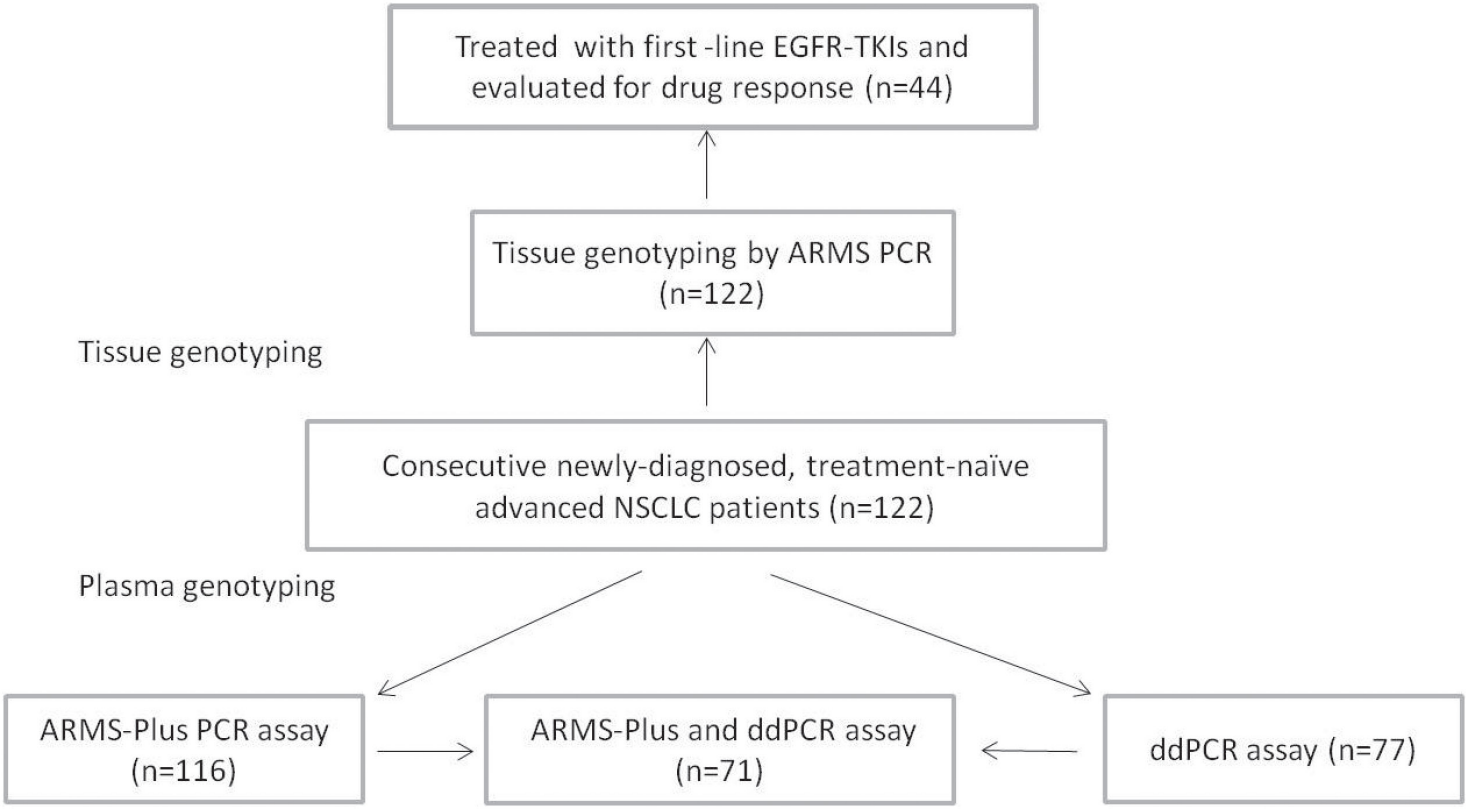
Flow chart of patient enrollment NSCLC, non-small cell lung cancer; EGFR-TKI, epidermal growth factor receptor-tyrosine kinase inhibitor.

Sensitizing EGFR mutations were positive in 45 (36.9%) patients and negative in the remaining 77 (63.1%) patients. Plasma samples of 116 and 77 patients were tested by ARMS-Plus and ddPCR, respectively. Follow-up assessment was conducted in 44 patients who received EGFR-TKI therapy (Figure 3).

### Diagnostic performance of ARMS-Plus

For the 116 patients with plasma samples analyzed by ARMS-Plus, 44 (37.9%) of them were harboring EGFR activating mutations in tumor tissue (Table 2). Among them, 23 patients with 19del and 11 patients with L858R were correctly identified by ARMS-Plus, yielding an overall sensitivity of 77.27% (34/44; 95% CI: 61.78%-88.01%). Detection sensitivity for 19del and L858R were 79.31% (23/29) and 68.75% (11/16), respectively. Using tumor tissue EGFR mutation as the gold standard, false-negative results were found in 10 patients, including 5 with 19del, 4 with L858R and one with both 19del and L858R mutations. Two cases of false-positive were found and thus the overall specificity for ARMS-Plus was 97.22% (70/72; 95% CI: 89.42%-99.52%). The corresponding specificity for 19del and L858R were 98.85% (86/87) and 99.00% (99/100), respectively. The concordance rate of the EGFR mutations detected in tissue and plasma specimens was 89.66% (104/116; κ=0.77, *P*<0.0001). The positive predictive value was 94.44% (95% CI, 79.99%-99.03%).

**Table 2:** Performance of ARMS-Plus and ddPCR for the detection of EGFR mutations in plasma

		ARMS-Plus		ddPCR	
		**%**	**n**	**%**	**n**
Sensitivity	**Overall**	77.27	34/44	72.00	18/25
	**19del**	79.31	23/29	62.50	10/16
	**L858R**	68.75	11/16	88.89	8/9
Specificity	**Overall**	97.22	70/72	100	52/52
	**19del**	98.85	86/87	100	61/61
	**L858R**	99.00	99/100	100	68/68
		% (κ)	n	% (κ)	n
Concordance^*^	**Overall**	89.66 (0.77)	104/116	90.91 (0.78)	70/77
	**19del**	93.97 (0.83)	109/116	92.21 (0.73)	71/77
	**L858R**	94.83 (0.76)	110/116	98.70 (0.93)	76/77

^*^All results in concordance had a *P* value of less than 0.0001.

### Diagnostic performance of ddPCR

25 of the 77 (32.5%) patients tested with ddPCR were positive for EGFR activating mutations in the paired tumor tissue. Among them, 18 patients with either 19del or L858R mutation were correctly identified by ddPCR. False-negative cases occurred in 7 patients, while no false-positive results were found (Table 2). Collectively, the overall concordance rate between plasma ddPCR and tissue genotyping was 90.91% (70/77). The sensitivity and specificity for EGFR mutation testing by ddPCR were 72.00% (18/25) and 100% (52/52), respectively. The positive predict value was 100% (95% CI, 78.12%-100.00%).

### Comparison of the performance of ARMS-Plus and ddPCR

Plasma samples from 71 patients were tested by both ARMS-Plus and ddPCR (Table 3). 24 of them (33.80%) were harboring either 19del or L858R mutation in the paired tumor tissue. The sensitivity, specificity, and concordance rate for ARMS-Plus were 83.33% (20/24), 100% (47/47) and 94.37% (67/71), respectively. While, the sensitivity, specificity, and concordance rate for ddPCR were 70.83% (17/24), 100% (47/47) and 90.14% (64/71), respectively. The performance of both ARMS-Plus and ddPCR within this subgroup of patients were consistent with that of the entire population in this study. The concordance rate between the two platforms was 92.96% (66/71). These suggested that the two assays were repeatable and comparable in detecting plasma EGFR mutations.

**Table 3:** A head-to-head comparison of the performance of ARMS-Plus and ddPCR in a subset of 71 patients tested by both assays

	Sensitivity	Specificity	Concordance	Kappa value (*P*)
ARMS-Plus	83.33% (20/24)	100% (47/47)	94.37% (67/71)	0.87 (<0.0001)
ddPCR	70.83% (17/24)	100% (47/47)	90.14% (64/71)	0.76 (<0.0001)

Among this subpopulation, a total of 8 cases of discordant results were found between tissue and plasma genotyping (Supplementary Table 1). In which, both ARMS-Plus and ddPCR had 3 cases of false-negative result. Notably, for the 5 cases where ARMS-Plus and ddPCR had a contrasting result, ARMS-Plus correctly identified the EGFR mutation in 4 cases, while ddPCR only correctly identified the mutation in one case.

### Correlation between EGFR mutation status and EGFR-TKIs efficacy

44 TKI-naïve patients with positive tumor tissue EGFR activating mutations had received EGFR-TKIs. The first 8-week follow-up was completed and the patients’ response to EGFR-TKIs was evaluated (Supplementary Table 2). According to the plasma EGFR mutation status, patients were divided into two subgroups: patients with EGFR mutations detected in both tissue and plasma specimens (T+P+, n=34), and patients with tumor tissue EGFR mutations only (T+P-, n=10). The objective response rate (ORR) was 44.1% (15/34) and 30.0% (3/10) for the T+P+ group and T+P- group, respectively. The disease control rate (DCR) of the two groups was (94.1%, 32/34) and (90.0%, 9/10), respectively. Although there seemed to be a trend of better ORR in the T+P+ group, no significant differences in both ORR and DCR were observed between the two groups (*P*=0.489 and 0.548).

### Influencing factors that affect diagnostic performance of ARMS-Plus

The total plasma circulating DNA concentration, represented by the wild-type EGFR alleles in our research, has been reported to affect detection sensitivity [17]. To verify this, we compared the wild-type EGFR allele concentration between the T+P+ and T+P- groups. The median of total plasma wild-type EGFR allele concentration was 3676.8/mL and 2098.0/mL for the T+P+ and T+P- groups, respectively (Supplementary Figure 2). No significant difference was observed between the two groups (*P*=0.1).

Besides, it has been noticed that metastasis has an effect upon plasma genotyping [8]. Indeed, we found that the sensitivity of ARMS-Plus for the detection of EGFR activating mutations increased with the increase of number of metastatic sites (*P*=0.013) (Supplementary Figure 3).

## DISCUSSION

Tissue biopsy as the current standard diagnostic procedure possesses a high reliability, yet, the success rate is dismal. It is estimated that 10% to 50% of patients who underwent biopsy failed to obtain sufficient tumor tissues for EGFR genotyping [10]. Even in well-designed prospective clinical trials [2, 18], the success rate of bronchial biopsy was less than 50%[19]. To overcome the limitations of tissue biopsies, a non-invasive, quantitative, and real-time blood-based assay for the detection of EGFR activating mutations is urgently warranted.

According to previous studies, the sensitivity, specificity, and concordance rate for QIAGEN therascreen EGFR RGQ PCR Kit (an ARMS-based technique) in detecting plasma EGFR mutations were 45.4–65.7%, 90–99%, and 78–95%, respectively [5, 11]. To solve the problem of low sensitivity, we optimized the primer design and employed a “Wild-type blocker” in order to strengthen the selective amplification of low-abundance EGFR mutations. As a result, ARMS-Plus had an improved sensitivity (77.27%) for detecting plasma EGFR mutations, while maintaining a high specificity (97.22%).

ddPCR has been demonstrated to be a rapid and reliable method for plasma genotyping [8, 14, 20, 21]. Our results showed that the sensitivity and specificity of ddPCR were 72.00% (18/25) and 100% (48/48), respectively, which is consistent with the results of a previous study, reporting a sensitivity and specificity of 74.1% and 100% [21].

To evaluate the detection efficiency of ARMS-Plus and ddPCR, we also conducted a prospective head-to-head comparison between the two detection platforms. A total of 71 plasma samples were tested by both assays. In this analysis, ARMS-Plus showed a higher sensitivity than ddPCR in detecting plasma EGFR mutations (83.33% versus 70.83%). High detection specificity was observed in both assays. The concordance rate of the two platforms was 92.96% (66/71), indicating that the performance of ARMS-Plus and ddPCR for the detection of EGFR mutations in plasma samples were comparable. In addition, 5 discordant cases were found between the tumor tissue and plasma genotyping, in which ARMS-Plus correctly identified the EGFR mutant alleles in 4 patients, while ddPCR was only correct in one case. This implied that ARMS-Plus was more sensitive than ddPCR in certain patients. Besides, there were 3 cases of false-negative for both ARMS-Plus and ddPCR, suggesting that the two assays had several common weaknesses in plasma genotyping.

We also analyzed the possible causes of discrepancy between the results of tumor tissue and plasma genotyping. Other studies revealed that false-negative results were mainly due to low mutation rate in plasma samples [14, 21]. Hence, we compared the total cfDNA levels between the T+P+ and T+P- groups for the 11 false-negative cases in our study. No significant difference in plasma total wild-type EGFR allele concentration was found between the two groups (*P*=0.34), indicating that the false-negative results in our system were unlikely to be caused by low mutation rate. The dynamic change of ctDNA level could be a possible explanation for the incidence of false-negative cases [22]. As for the 2 false-positive cases, we reanalyzed the paired tumor tissues with Sanger sequencing for further validation. Results were still negative, however, considering the low sensitivity of direct sequencing, we cannot rule out the possibility of the presence of low frequency mutation in the tumor tissues. Moreover, previous research suggested that intratumoral genetic heterogeneity does exist in lung cancer [23]. Mutation status of the same tumor can be different in two spatially separated regions. Therefore, a negative result of a captured tissue sample cannot represent the absence of EGFR mutations in the entire tumor [12, 24].

To investigate the correlation between plasma EGFR mutation status and EGFR-TKI efficacy, we assessed the clinical outcomes of patients received EGFR-TKIs. Although no significant differences were observed in both ORR (*P*=0.489) and DCR (*P*=0.548) between the T+P+ and T+P- groups, the T+P+ group showed a considerably better ORR than the T+P- group (44.1% versus 30.0%). This is consistent with a previous study, reporting that patients with both tumor tissue and plasma EGFR activating mutations showed superior progression-free survival (PFS) and overall survival (OS) compared to patients with tumor tissue mutations only [10, 20].

Also, it has been reported that the sensitivity of plasma-based genotyping was associated with tumor burden [8]. To validate this claim, we analyzed the sensitivity for ARMS-Plus to detect plasma EGFR mutations in patients with a different number of metastatic sites. Our results indicated that the sensitivity of ARMS-Plus increased with the increase of number of metastatic sites (*P*=0.013), supporting the hypothesis that heavy tumor burden leads to higher ctDNA levels in blood and thus facilitates plasma-based genotyping.

**Figure 4:**
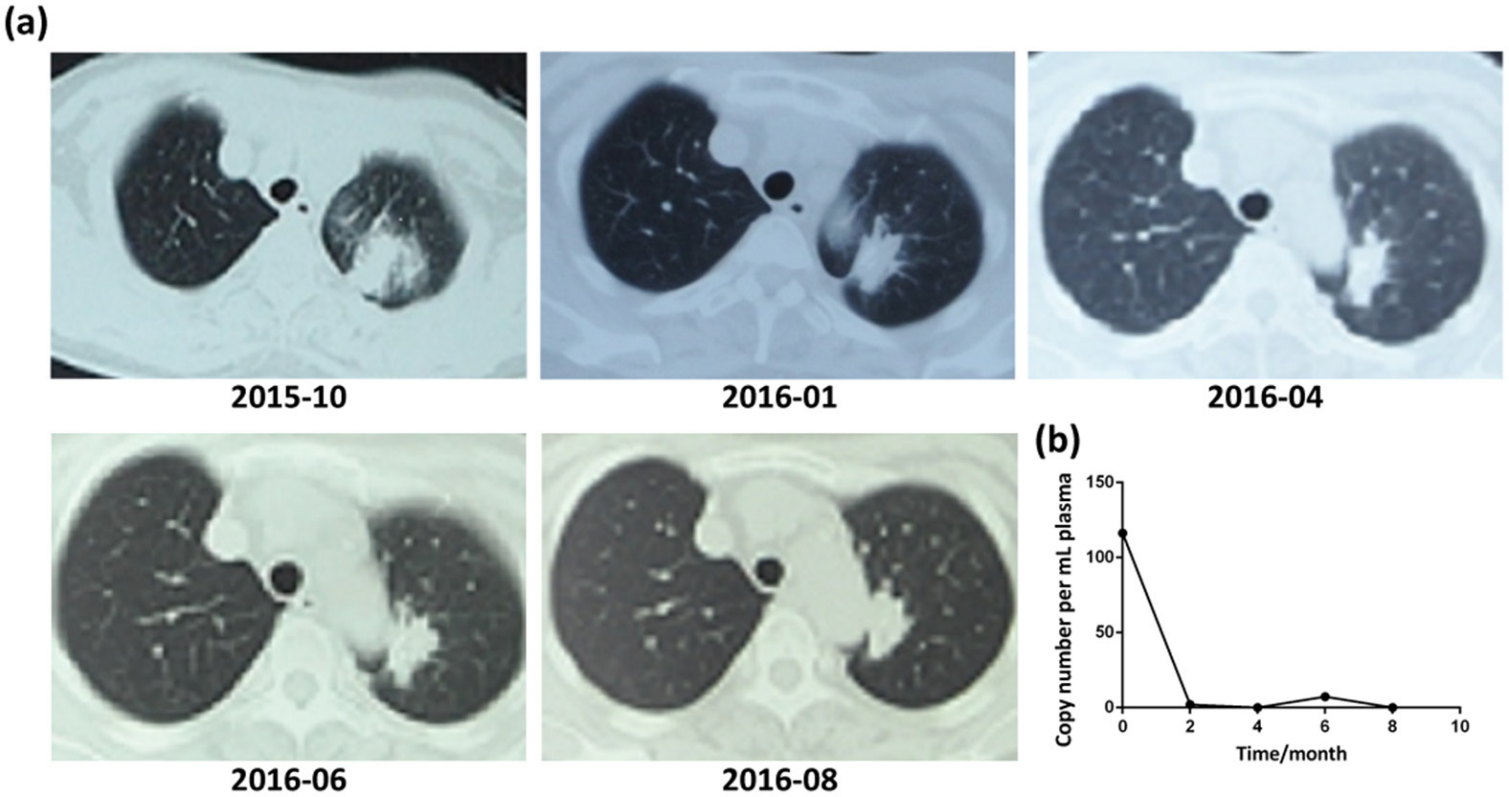
Correlation between radiological responses and the concentration of EGFR mutant alleles in plasma: a case report of a T+P+ patient **(a)** Serial CT images of the patient. The diagnosis was made at 2015-10 and responses to gefitinib were evaluated every two months thereafter. **(b)** Longitudinal monitoring of plasma EGFR 19del concentration using ARMS-Plus.

Given the non-invasive nature of liquid biopsies, plasma genotyping methods have been investigated for its ability to monitor treatment response [21, 25]. In this study, change in the concentration of EGFR mutant alleles was assessed by ARMS-Plus in several cases. Herein, we reported a typical T+P+ case of a patient with notable clinical and radiological responses to EGFR-TKI therapy. A 58 years old woman (never-smoker, ECOG PS 1) was diagnosed with stage IV lung adenocarcinoma, harboring both tumor tissue and plasma EGFR 19del mutation. After receiving gefitinib for 2 months, the primary lesion in the left lung significantly shrunk in size according to CT imaging (Figure 4). Partial response (PR) was achieved. Since the initiation of gefitinib therapy, the size of the lesion remained unchanged for up to 10 months. Meanwhile, the plasma concentration of EGFR 19del alleles detected by ARMS-Plus also remained at a low level, which is in agreement with the radiological findings. This suggested that ARMS-Plus could be a potential assay for dynamic monitoring of therapeutic responses. The application of ARMS-Plus in disease progression management requires further investigation.

To sum up, our research demonstrated that ARMS-Plus possessed a high sensitive and specificity for the detection of EGFR activating mutations in advanced NSCLC patients, with a diagnostic performance comparable to that of ddPCR. Considering that ddPCR demands specialized detection and analyzing equipment while ARMS-Plus could be conveniently performed with a real-time PCR device, ARMS-Plus could be a more effective method for detecting plasma EGFR activating mutations when tissue genotyping is not available in clinical practice.

## MATERIALS AND METHODS

### Study design

Newly-diagnosed, treatment-naïve advanced NSCLC patients were enrolled consecutively in this plasma genotyping study (NCT02666755) at the Xijing hospital from November 2015 to June 2016. The project was approved by the Ethics Committee of the Fourth Military Medical University (20130121-6). Inclusion criteria include: age ranged from 18-85 years old; stage III to IV NSCLC patients; with at least one specific measurable lung cancer lesions (diameter 10 mm or longer); without severe hemorrhagic disease. Signed informed consent to participate in the study was obtained from all the patients. EGFR-TKIs were administrated according to their tissue genotyping results. Clinical response, in terms of ORR and DCR, was assessed after 8 weeks of treatment on the basis of computed tomography (CT) scans according to the Response Evaluation Criteria in Solid Tumors (RECIST) 1.1.

### Specimen collection and DNA extraction

Tissue samples of most patients were derived from the primary tumor, except for only one case of brain metastasis. Samples used for tissue genotyping were mostly fresh biopsy tumor tissues, formalin-fixed paraffin-embedded (FFPE) tissues were used in 5 cases. One day after tissue sampling, 10 mL blood was collected in tubes containing trisodium citrate. Blood sample was centrifuged at 2500 g for 10 min at 4°C within 2 hours after collection. The plasma supernatant was isolated and stored at −20°C for no more than 2 weeks until delivery for further processing and genotyping. 2 mL plasma samples (where available) were assigned for each platform assessment and were tested in a blinded fashion.

Plasma samples were delivered in dry ice to GenoSaber Biotech Co., Ltd., Shanghai and Amoy Diagnostics Co., Ltd., Xiamen for ARMS-Plus and ddPCR, respectively within 2 days. ctDNA of NSCLC patients and cfDNA from healthy individuals were extracted from the plasma samples by using QIAamp Circulating Nucleic Acid kit (Qiagen, Hilden, Germany) according to the manufacturer’s instruction. Genomic DNA was extracted using QIAmp DNA Minikit (QIAGEN, CA, USA) from fresh tumor samples or with FFPE DNA kit (Amoy Diagnostics Co., Ltd., Xiamen, China) from FFPE tissues.

### Detection of EGFR mutations by ARMS-Plus

ARMS-Plus was conducted with Human *EGFR* Gene Mutation Quantitative Detection Kit (Fluorescence qPCR) (Genosaber Biotech, Shanghai, China) at Genosaber Biotech in Shanghai. Briefly, the reaction mixture in 0.2mL thin well 6-tube strip was melted at room temperature and centrifuged at 3000g for 1 min. Then 5μl samples or control materials came with the kit were added to the 45μl reaction mixture. Before loading, strips containing samples, control materials and calibrators (pre-mixed) were vortexed for 10-20s and centrifuged at 3000g for 4 min. All three types of mutation of the same sample were analyzed in the same array in order to standardize the detection conditions, with an external control at the conserved region of EGFR exon 4 to monitor sample qualities. PCR was conducted with Applied Biosystems^®^ 7500 Real-Time PCR Systems (Applied Biosystems, USA). The thermocycling conditions were: hot start at 95°C for 4 min, and 50 cycles of 95°C for 10 s, 61°C for 30s with fluorescence reading (FAM). Analyzed data was processed using the program came with Applied Biosystems^®^ 7500 Real-Time PCR Systems. Copy number and mutation frequency were calculated. Detailed procedures can be seen in supplementary material (Supplementary Table 3).

### Detection of EGFR mutations by ARMS PCR and ddPCR

Tissue genotyping of 21 types of EGFR mutations with ARMS PCR was conducted using Human EGFR Gene Mutations Fluorescence Polymerase Chain Reaction (PCR) Diagnostic Kit (Amoy Diagnostics Co., Ltd., Xiamen, China) according to the manufacturer’s instructions [23]. Detection and quantification of EGFR mutations by ddPCR (Amoy Diagnostics Co., Ltd., Xiamen, China) was conducted as previously described [13].

### Statistical analysis

The sensitivity and specificity of the blood test (detected by ddPCR and ARMS-Plus) were calculated by comparing with the paired tumor tissue result (detected by ARMS PCR). The CIs for sensitivity and specificity were computed with the Clopper and Pearson method. The consistency between blood and tissue was assessed by Cohen’s k test. Wide-type EGFR concentration of T+P+ and T+P- groups were compared using Mann-Whitney Test. Pearson correlation analysis was employed to assess the relationship between the number of metastasis and detection sensitivity. The correlation between clinical responses and plasma EGFR mutation status was measured by Chi-square test or Fisher’s exact test. P<0.05 was considered significant. All tests were two-sided. Statistical analyses and data visualization were performed using SPSS 19.0 and GraphPad 6.0.

## SUPPLEMENTARY MATERIALS FIGURES AND TABLES


