# Supplementary material for: A comparison of ARMS-Plus and droplet digital PCR for detecting EGFR activating mutations in plasma
Source: Oncotarget. 2017 Dec 6;8(67):112014–23. doi: 10.18632/oncotarget.22997 (PMC5762375; doi:10.18632/oncotarget.22997)
Supplement: Supplementary file 1 [file oncotarget-08-112014-s001.pdf]

A comparison of ARMS-Plus and droplet digital PCR for detecting EGFR activating mutations in plasma

SUPPLEMENTARY MATERIALS

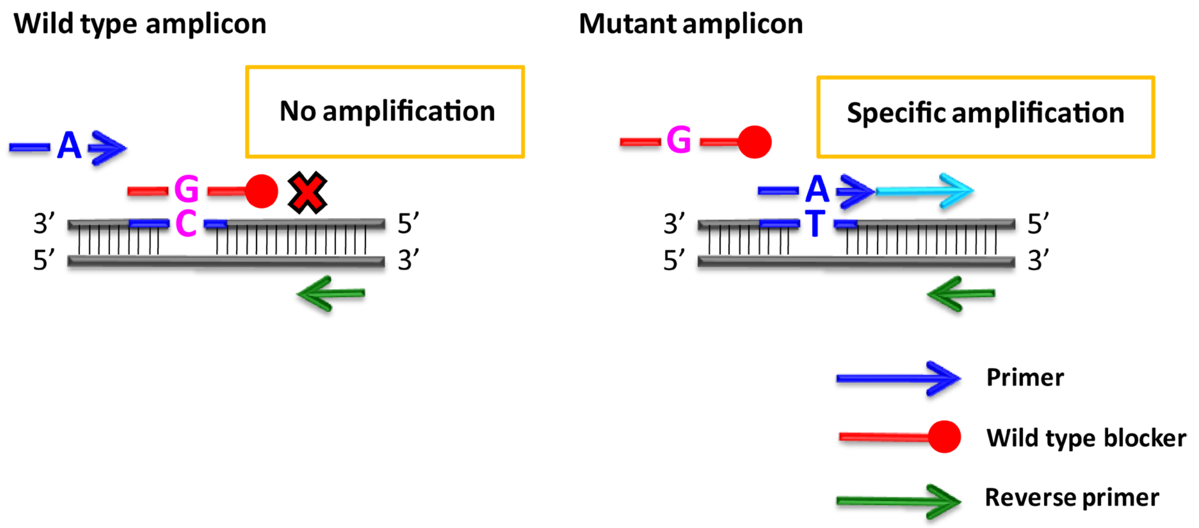

Supplementary Figure 1: Schematic representation of the principle of ARMS-Plus. When the concentration of wild-type DNA is high, non-specific binding of primers to wild-type DNA may occur, leading to false-positive results. The “Wild-type blocker” in ARMS-Plus could specifically bind to wild-type DNA and prevent the false-positive amplification, resulting in a precise detection of the target mutant sequence.

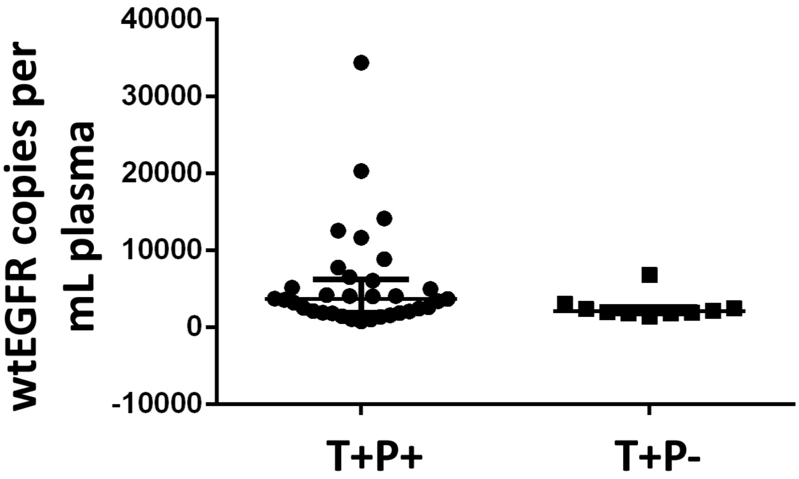

Supplementary Figure 2: Comparison of the wild-type EGFR abundance in plasma between T+P+ and T+P- groups. To evaluate the influence of background DNA to the detection, the wild-type EGFR abundance was compared between T+P+ and T+P- groups. The median total plasma wild-type EGFR allele concentration was 3676.8 copies/mL and 2098.0 copies/mL for T+P+ and T+P- groups, respectively. No significant difference was observed between the two groups (*P*=0.1).

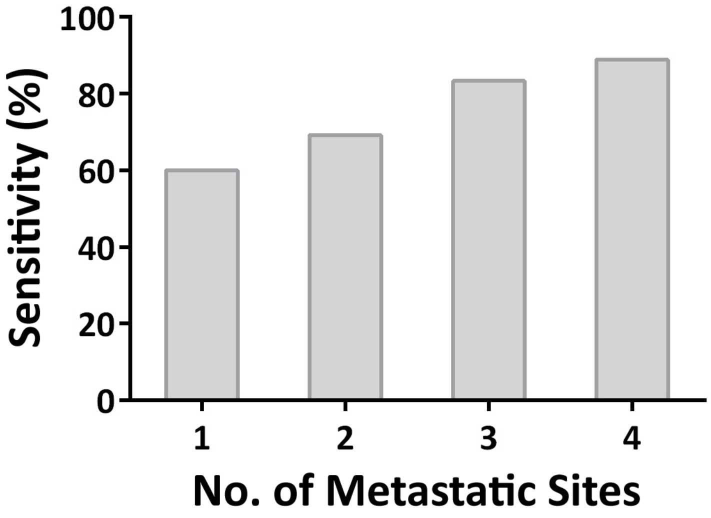

Supplementary Figure 3: Association between the sensitivity of ARMS-Plus and the number of metastasis. The sensitivity of ARMS-Plus for the detection of EGFR activating mutations increases along with the number of metastasis (*P*=0.013).

Supplementary Table 1: Summary of the discordant cases between tissue and plasma genotyping

Case No.	Gender	Age/ Years	Histology subtype	Stage	tissue	ddPCR	ARMS-Plus
N001	M	65	Adenocarcinoma	IV	19del	WT	19del
N002	F	50	Adenocarcinoma	IV	19del	WT	19del
N003	F	56	Adenocarcinoma	IV	19del	WT	19del
N004	F	48	Adenocarcinoma	IV	L858R	WT	L858R
N005	M	51	Adenocarcinoma	IV	L858R	L858R	WT
N006	M	54	Adenocarcinoma	IV	19del	WT	WT
N007	M	59	Adenocarcinoma	IV	19del	WT	WT
N008	F	58	Adenocarcinoma	IV	19del	WT	WT

Supplementary Table 2: Correlation between plasma EGFR mutation status and EGFR-TKI efficacy

Group	CR	PR	SD	PD
T^+P^+	2	13	17	2
T^+P^-	0	3	6	1

T^+P^+: Patients harboring mutations in both tissue and plasma specimens;
T^+P^-: Patients harboring mutations in tissue specimen only;
CR, complete response; PR, partial response; SD, stable disease; PD, progress disease.

Supplementary Table 3: Sequence of primers and probes for ARMS-Plus

	Forward primer	Reverse primer	Probe	Blocker
**wild-type EGFR**	TGGAGAGCATCCAGT	TCTGGAAGTCCATCG ACAT	5'FAM-ACATAG TCAGCAGTG ACTT-3'MGB	none
**EGFR L858R**	AGATTTTGGGCG	TTTGCCTCCTTCTGC	5'FAM-AACTGCTGGGT GCGGA-3'MGB	TTTTGGGCGG GCCAAAC-3'end-phosphorothioated
**EGFR 19del 1**	CCGTCGCTATCAAA	ATGGACCCCCACAC	5'FAM-AAGCCAACAAG GAAAT-3'MGB	none
**EGFR 19del 2**	GTCGCTATCAAGA	CATGGACCCCCACA	5'FAM-AAGCCAACAAGGAAAT-3'MGB	none
